# Higher Serum Cholesterol Levels Are Associated With Reduced Systemic Inflammation and Mortality During Tuberculosis Treatment Independent of Body Mass Index

**DOI:** 10.3389/fcvm.2021.696517

**Published:** 2021-06-22

**Authors:** Vignesh Chidambaram, Lucas Zhou, Jennie Ruelas Castillo, Amudha Kumar, Samuel K. Ayeh, Akshay Gupte, Jann-Yuan Wang, Petros C. Karakousis

**Affiliations:** ^1^Division of Infectious Diseases, Department of Medicine, Johns Hopkins School of Medicine, Baltimore, MD, United States; ^2^Department of Internal Medicine, University of Arkansas for Medical Sciences, Little Rock, AR, United States; ^3^Department of Internal Medicine, National Taiwan University Hospital, Taipei, Taiwan; ^4^Department of International Health, Johns Hopkins Bloomberg School of Public Health, Baltimore, MD, United States

**Keywords:** LDL-cholesterol, HDL-cholesterol, inflammation, BMI, effect modification

## Abstract

**Background:** Lipids play a central role in the pathogenesis of tuberculosis (TB). The effect of serum lipid levels on TB treatment (ATT) outcomes and their association with serum inflammatory markers have not yet been characterized.

**Methods:** Our retrospective cohort study on drug-susceptible TB patients, at the National Taiwan University Hospital, assessed the association of baseline serum lipid levels such as low-density lipoprotein (LDL), high-density lipoprotein (HDL), total cholesterol (TC) and triglycerides (TG) with all-cause and infection-related mortality during first 9 months of ATT and baseline inflammatory markers namely C-reactive protein (CRP), total leukocyte count (WBC), and neutrophil-lymphocyte ratio (NL ratio).

**Results:** Among 514 patients, 129 (26.6%) died due to any-cause and 72 (14.0%) died of infection. Multivariable Cox-regression showed a lower adjusted hazard ratio (aHR) of all-cause mortality in the 3rd tertiles of HDL (aHR 0.17, 95% CI 0.07–0.44) and TC (aHR 0.30, 95% CI 0.14-0.65), and lower infection-related mortality in the 3rd tertile of HDL (aHR 0.30, 95% CI 0.14–0.65) and TC (aHR 0.30, 95% CI 0.14–0.65) compared to the 1st tertile. The 3rd tertiles of LDL and TG showed no association in multivariable analysis. Similarly, 3rd tertiles of HDL and TC had lower levels of baseline inflammatory markers such as CRP, WBC, and NL ratio using linear regression analysis. Body mass index (BMI) did not show evidence of confounding or effect modification.

**Conclusions:** Higher baseline serum cholesterol levels were associated with lower hazards of all-cause and infection-related mortality and lower levels of inflammatory markers in TB patients. BMI did not modify or confound this association.

## Introduction

Tuberculosis (TB) is an infectious disease with a high morbidity and mortality (1). Patients with TB have an increased incidence of cardiovascular (2) and cerebrovascular disease (3). Lipids play a central role in the pathogenesis of both TB and atherosclerotic vascular disease (4, 5). However, the effect of serum lipid levels on anti-tuberculosis treatment (ATT) outcomes is not yet characterized.

Studies have shown that TB patients have lower serum cholesterol levels compared to uninfected individuals (6–8). Sputum smear positivity for *Mycobacterium tuberculosis* (*Mtb*) was associated with lower serum levels of low-density lipoprotein (LDL), high-density lipoprotein (HDL) and total cholesterol (TC) (7). Similarly, lower levels of LDL, HDL and TC correlated with more radiologically extensive TB disease in the lungs (7, 8). Interestingly, this pattern also holds true for the association between lipid levels and community-acquired pneumonia (7). Lower lipid levels have also been linked to higher inflammation in critically ill patients following sepsis (9), pneumonia (10), and surgery (11). Whether inflammation plays an important role in the association between lipid levels and TB outcomes is unclear.

Low body mass index (BMI), an objective marker of low nutritional status in adults, is associated with increased mortality in TB patients (12, 13). Though serum lipid levels are directly related to BMI in the under-weight and normal-weight categories, there is an inverse association with higher BMI, resulting in an inverted U-shaped relationship between these two markers (14). Thus, it becomes important to assess whether nutritional status, as suggested by BMI, confounds, or modifies the association between serum lipid levels and inflammation, and mortality in TB.

We carried out a retrospective cohort study on pulmonary TB patients receiving standard antitubercular treatment to assess the association between serum lipid levels and all-cause and infection-related mortality during 9 months of ATT initiation. We also studied the association of lipid levels with baseline serum markers of systemic inflammation at TB diagnosis.

## Methods

### Design and Study Population

In our retrospective cohort on drug-susceptible TB patients (age >18 years), we used the National Taiwan University Hospital (NTUH) database from 2000 to 2016 (15, 16). TB diagnosis was confirmed by culture-based detection using MGIT-960 and Lowenstein-Jensen medium. Most patients were treated on an outpatient basis according to ATS guidelines (17). We included patients who were tested for at least one of the following serum lipid levels: LDL, HDL, TC, or triglycerides (TG), with no specific exclusion criteria. We collected baseline demographic data, such as age, sex, and comorbidities, including diabetes mellitus, hypertension, coronary artery disease, heart failure, transplantation (both solid organ and bone marrow transplantation), smoking, and alcohol use disorder. BMI at TB diagnosis was calculated as weight (kg) divided by the square of the height (m^2^). Data on baseline TB characteristics, such as sputum smear for acid-fast bacilli (AFB) grading (0 to 4+), prior history of TB, and presence of cavitary disease on chest radiography, were obtained. Charlson comorbidity index (CCI) was calculated from the variables obtained from the database (18). The study was approved by the institutional review boards at Johns Hopkins University and NTUH.

### Exposures

The exposures assessed in our study were serum lipids, namely LDL, HDL, TC, and TG. Patients were categorized into three groups based on tertiles of the baseline lipid levels, separately for each of the above-mentioned lipids. Baseline lipid levels were defined as a test performed anytime between 1 month before and 1 month after TB diagnosis.

### Inflammatory Markers

Serum inflammatory markers, namely C-reactive protein (CRP) (mg/dL), erythrocyte sedimentation rate (ESR) (mm/hr), total leukocyte count (WBC) (x 10^3^/μL) and neutrophil-lymphocyte ratio (NL ratio) at baseline were documented. Any test result for the inflammatory markers within 30 days of TB diagnosis was considered as baseline.

### Outcomes

The primary outcomes evaluated were all-cause and infection-related mortality during the first 9 months of ATT. The latter was a composite outcome of death due to TB, pneumonia, or sepsis.

### Statistical Analysis

Patient characteristics stratified by tertiles of the baseline serum lipid levels were compared using ANOVA for normally distributed data and Kruskal-Wallis test for non-normally distributed data, and Chi-square test for categorical variables. Kaplan-Meier and Cox proportional hazards methods were used to measure the association between the lipid tertiles and all-cause and infection-related mortality in separate models. Person-time at risk of outcome, for each of the lipid tertiles, was calculated from the time of ATT initiation until 9 months, or loss to follow-up or death, whichever occurred first. Point of loss to follow-up was defined as the last study visit prior to 9 months. Potential confounders for the multivariable analyses were identified by literature review and by exploratory univariable data analysis at *p* < 0.05 significance. Confounding factors that are components of the CCI were not adjusted for separately if CCI was included in the multivariable model. We performed sensitivity analyses after excluding patients who died within the first month of ATT since the definition for “baseline lipid levels” included up to 1 month after TB diagnosis. Sensitivity analysis was also performed using the exposure defined as mean value of all available lipid test results during the 9-month period after ATT initiation or the duration of follow-up, if lost to follow-up or death occurred before 9 months.

Association of baseline serum lipid levels and inflammatory markers, namely CRP, ESR, WBC and NL ratio, were analyzed using univariable and multivariable linear regression analyses. We performed sensitivity analysis by assessing the association of baseline lipid levels and inflammatory markers obtained up to day 15 of ATT to account for the possibility of a rapid decrease in inflammation after ATT initiation.

The association of tertiles of baseline lipid levels with BMI was assessed using linear regression analysis. Effect modification by BMI at different cut-offs, on the association between lipid levels and mortality or elevated inflammatory markers were evaluated using the Cox and logistic regression models, respectively, by stratification, multiplicative and additive interactions. Relative excess of risk index (RERI) was used to assess the magnitude and the significance of the additive effect modification using the “ic package” in STATA. All analyses were performed using Stata, version 16.0 IC (StataCorp LP, College Station, TX).

## Results

### Baseline Characteristics Stratified by Lipid Tertiles

In our cohort, among 2,894 patients with drug-sensitive pulmonary TB, 514 patients with at least one serum lipid measurement available at baseline were included (Supplementary Figure 1). The median (IQR) range was 69.8 years (53.9–78.8), and 384 patients (74.7%) were male (Table 1). Forty-three percent of this population were ever-smokers, and 2.7% reported alcohol use disorder. At baseline, 221 patients (43.2%) had positive sputum AFB smears and 14% patients had cavitation on chest imaging. Nineteen patients (7.2%) had a past history of TB.

**Table 1:** Patient characteristics based on tertiles of baseline LDL and HDL levels.

**Study characteristics**	**Measure**	**All patients (*N* = 514)**	**LDL**				**HDL**			
			**LDL tertile 1 (*n* = 92)**	**LDL tertile 2 (*n* = 90)**	**LDL tertile 3 (*n* = 88)**	***p*-value**	**HDL tertile 1 (*n* = 83)**	**HDL tertile 2 (*n* = 86)**	**HDL tertile 3 (*n* = 80)**	***p*-value**
Age (years)	Median (IQR)	69.8 (53.9–78.8)	70.8 (52.8–77.7)	71.4 (55.1–77.9)	68.4 (56.9–76.9)	0.879	71.8 (53.2–77.8)	70.6 (56.7–78.2)	68.6 (55.3–76.3)	0.581
Male Sex	No (%)	384 (74.7%)	65 (70.7%)	72 (80%)	66 (75%)	0.344	74 (81.3%)	56 (71.8%)	56 (70%)	0.183
BMI	Mean (SD)	21.4 (3.7)	19.9 (3.7)	22.5 (4.3)	22.4 (3.1)	<0.001	20.6 (3.8)	22.4 (3.6)	21.6 (3.7)	0.024
Initial AFB smear positivity	No. (%)	221 (43.2%)	43 (46.7%)	43 (48.3%)	39 (44.8%)	0.898	48 (53.3%)	35 (44.9%)	34 (43.0%)	0.355
Initial AFB smear grade	Median (IQR)	0 (0–2)	0 (0–2)	0 (0–2)	0 (0–2)	0.816	1 (0–2)	0 (0–2)	0(0–2)	0.619
Prior TB	No. (%)	19 (7.2%)	5 (10.2%)	2 (4.2%)	3 (7.9%)	0.520	5 (10.2%)	2 (4.2%)	3 (7.9%)	0.520
Cavitary disease	No. (%)	72 (14.0%)	13 (14.1%)	11 (12.2%)	17 (19.3%)	0.395	6 (12.2%)	0 (0%)	3 (8.1%)	0.090
Smoking	No. (%)	117 (43.4%)	32 (48.5%)	31 (43.1%)	42 (51.9%)	0.551	38 (58.5%)	31 (50.8%)	30 (41.1%)	0.123
Alcoholism	No. (%)	14 (2.7%)	1 (1.1%)	2 (2.2%)	2 (2.3%)	0.799	4 (4.4%)	1 (1.3%)	0 (0%)	0.106
DM	No. (%)	141 (27.4%)	25 (27.2%)	30 (33.3%)	35 (39.7%)	0.201	25 (27.5%)	26 (33.3%)	26 (32.5%)	0.666
HTN	No. (%)	263 (51.2%)	51 (55.4%)	45 (50.0%)	46 (52.3%)	0.762	50 (54.9%)	38 (48.7%)	41 (51.3%)	0.716
Cancer	No. (%)	81 (15.8%)	15 (16.3%)	11 (12.2%)	8 (9.1%)	0.342	8 (8.8%)	9 (11.5%)	15 (18.8%)	0.139
CKD Stage ≥ 3	No. (%)	37 (7.2%)	13 (14.1%)	4 (4.4%)	2 (2.27%)	0.004	8 (8.8%)	6 (7.7%)	4 (5.0%)	0.622
Asthma	No. (%)	21 (4.1%)	4 (4.4%)	1 (1.1%)	7 (7.9%)	0.086	4 (4.4%)	2 (2.6%)	5 (6.3%)	0.530
COPD	No. (%)	88 (17.1%)	11 (11.9%)	19 (21.1%)	17 (19.3%)	0.225	10 (10.9%)	18 (23.1%)	13 (16.3%)	0.107
Bronchiectasis	No. (%)	16 (3.1%)	2 (2.2%)	2 (2.2%)	3 (3.4%)	0.842	2 (2.2%)	2 (2.6%)	3 (3.8%)	0.818
Pneumoconiosis	No. (%)	4 (0.8%)	1 (1.1%)	1 (1.1%)	0 (0%)	0.614	0 (0%)	1 (1.3%)	0 (0%)	0.333
Autoimmune disease	No. (%)	25 (4.9%)	2 (2.2%)	3 (3.3%)	4 (4.6%)	0.675	3 (3.3%)	4 (5.1%)	1 (1.3%)	0.384
Liver cirrhosis	No. (%)	13 (2.5%)	4 (4.4%)	2 (2.2%)	0 (0%)	0.141	3 (3.3%)	2 (2.6%)	0 (0%)	0.282
History of transplant	No. (%)	16 (3.1%)	5 (5.4%)	5 (5.6%)	4 (4.6%)	0.946	2 (2.2%)	1 (1.3%)	10 (12.5%)	0.002
HIV	No. (%)	21 (4.1%)	9 (9.8%)	6 (6.7%)	1 (1.1%)	0.046	10 (10.9%)	2 (2.6%)	3 (3.8%)	0.042
CAD	No. (%)	72 (14.0%)	14 (15.2%)	19 (21.1%)	11 (12.5%)	0.281	13 (14.3%)	9 (11.5%)	15 (18.8%)	0.436
AMI	No. (%)	29 (5.64%)	6 (6.5%)	7 (12.5%)	3 (5.4%)	0.411	5 (5.5%)	6 (7.7%)	5 (6.3%)	0.842
CHF	No. (%)	35 (6.8%)	9 (15.5%)	9 (10.0%)	3 (3.4%)	0.211	9 (9.9%)	6 (7.7%)	7 (8.8%)	0.881
CVA	No. (%)	33 (6.4%)	9 (9.8%)	6 (6.7%)	4 (4.6%)	0.384	10 (10.9%)	5 (6.4%)	4 (5.0%)	0.398
CCI	Mean (SD)	4.4 (2.6)	5.2 (2.9)	4.9 (2.6)	4.1 (2.0)	0.029	4.7 (2.5)	4.6 (2.6)	4.5 (2.5)	0.818
Metformin use	No. (%)	81 (15.8%)	11 (11.9%)	20 (22.2%)	20 (22.7%)	0.112	10 (10.9%)	17 (21.8%)	14 (17.5%)	0.161
Statin use	No. (%)	61 (11.9%)	12 (13.0%)	19 (21.1%)	14 (15.9%)	0.335	10 (10.9%)	11 (14.1%)	16 (20.0%)	0.249
CCB use	No. (%)	117 (22.8%)	26 (28.3%)	20 (22.2%)	19 (21.6%)	0.510	23 (25.3%)	17 (21.8%)	21 (26.3%)	0.790

*AFB, acid–fast bacilli; AMI, acute myocardial infarction; BMI, body mass index; CAD, coronary artery disease; CCB, calcium channel blocker; CCI, charlson comorbidity index; CHF, congestive heart failure; CKD, chronic kidney disease; COPD, chronic obstructive pulmonary disease; CVA, cerebrovascular accident; DM, diabetes mellitus; HDL, high density lipoprotein cholesterol; HIV, human immunodeficiency virus; HTN, hypertension; IQR, interquartile range; LDL, low density lipoprotein cholesterol; PVD, peripheral vascular disease; SD, standard deviation*.

Patient characteristics stratified by tertiles of baseline serum lipid levels are shown in Tables 1, 2. Patients in the lowest LDL tertile had higher proportions of CKD Stage 3–5 (14.1 vs. 4.4 vs. 2.2%, *p*-value = 0.004) and higher mean values of CCI (5.2 vs. 4.9 vs. 4.1, *p*-value = 0.029). Patients in the lowest HDL tertile had a higher HIV proportion compared to the higher tertiles (10.9 vs. 2.6 vs. 3.8%, *p*-value = 0.004). There was a lower proportion of diabetes mellitus in the lowest tertiles of TC (12.2 vs. 35.7 vs. 27.9%, *p*-value = 0.025) and TG (18.7 vs. 28.3 vs. 35.6%, *p*-value = 0.003) (Tables 1, 2). Inflammatory markers, namely CRP, ESR, WBC and NL ratio, were available for 239 (46.4%), 27 (5.3%), 365 (71.0%) and 343 (66.7%) patients, respectively.

**Table 2:** Patient Characteristics based on tertiles of baseline Total Cholesterol (TC) and Triglycerides (TG) levels.

**Study Characteristics**	**Measure**	**Total Cholesterol (TC)**				**Triglycerides (TG)**			
		**TC tertile 1 (*n* = 146)**	**TC tertile 2 (*n* = 140)**	**TC tertile 3 (*n* = 143)**	***p*–value**	**TG tertile 1 (*n* = 166)**	**TG tertile 2 (*n* = 166)**	**TG tertile 3 (*n* = 163)**	***p*-value**
Age (years)	Median (IQR)	72.6 (54.1–81.2)	69.4 (53.4–78.7)	65.9 (51.9–76.4)	0.067	71.8 (54.1–81.5)	71.5 (56.7–79.2)	64.5 (51.9–76.8)	0.044
Male Sex	No (%)	109 (74.7%)	99 (70.7%)	107 (74.8%)	0.676	132 (79.5%)	119 (71.7%)	119 (73.0%)	0.214
BMI	Mean (SD)	19.9 (3.2)	22.3 (3.9)	22.2 (3.5)	<0.001	20.5 (3.5)	21.6 (3.6)	22.1 (3.6)	0.002
Initial AFB smear positivity	No. (%)	62 (42.5%)	64 (46.0%)	64 (45.1%)	0.820	63 (38.2%)	65 (39.2%)	79 (48.8%)	0.101
Initial AFB smear grade	Median (IQR)	0 (0–2)	0 (0–2)	0 (0–2)	0.538	0 (0–1)	0 (0–2)	0 (0–2)	0.126
Prior TB	No. (%)	11 (12.6%)	5 (7.3%)	3 (4.6%)	0.186	6 (6.9%)	5 (5.6%)	5 (6.3%)	0.940
Cavitary disease	No. (%)	18 (12.3%)	23 (16.4%)	23 (16.1%)	0.555	23 (13.9%)	19 (11.5%)	28 (17.2%)	0.326
Smoking	No. (%)	52 (52.0%)	45 (38.5%)	47 (38.5%)	0.072	55 (41.4%)	56 (41.5%)	63 (50%)	0.278
Alcoholism	No. (%)	3 (2.1%)	4 (2.9%)	5 (3.5%)	0.758	3 (1.8%)	2 (1.2%)	9 (5.5%)	0.038
DM	No. (%)	31 (12.2%)	50 (35.7%)	40 (27.9%)	0.025	31 (18.7%)	47 (28.3%)	58 (35.6%)	0.003
HTN	No. (%)	72 (49.3%)	61 (43.6%)	78 (54.6%)	0.182	68 (40.9%)	66 (39.8%)	76 (46.6%)	0.405
Cancer	No. (%)	18 (12.3%)	21 (15%)	22 (15.4%)	0.720	34 (20.5%)	22 (13.3%)	20 (12.3%)	0.077
CKD Stage ≥ 3	No. (%)	14 (9.6%)	7 (5%)	8 (5.6%)	0.240	14 (8.4%)	6 (3.6%)	16 (9.8%)	0.075
Asthma	No. (%)	4 (2.7%)	2 (1.4%)	10 (7%)	0.035	5 (3.0%)	8 (4.8%)	8 (4.9%)	0.627
COPD	No. (%)	22 (15.1%)	23 (16.4%)	29 (20.3%)	0.479	26 (15.7%)	32 (19.3%)	25 (15.3%)	0.567
Bronchiectasis	No. (%)	5 (3.4%)	4 (2.9%)	5 (3.5%)	0.947	2 (1.2%)	10 (6.0%)	3 (1.8%)	0.021
Pneumoconiosis	No. (%)	1 (0.7%)	1 (0.7%)	2 (1.4%)	0.777	0 (0%)	2 (1.2%)	2 (1.2%)	0.361
Autoimmune disease	No. (%)	4 (2.7%)	3 (2.1%)	8 (5.6%)	0.238	6 (3.6%)	5 (3.0%)	12 (7.4%)	0.128
Liver cirrhosis	No. (%)	7 (4.8%)	3 (2.1%)	2 (1.4%)	0.183	8 (4.8%)	4 (2.4%)	1 (0.6%)	0.057
History of transplant	No. (%)	3 (2.3%)	8 (7.2%)	4 (4.1%)	0.168	4 (2.4%)	7 (4.2%)	5 (3.1%)	0.642
HIV	No. (%)	9 (6.8%)	3 (2.7%)	3 (3.1%)	0.228	2 (1.2%)	10 (6.0%)	8 (4.9%)	0.067
CAD	No. (%)	18 (13.5%)	22 (19.8%)	12 (12.2%)	0.248	20 (12.1%)	29 (17.5%)	20 (12.3%)	0.273
AMI	No. (%)	6 (4.1%)	10 (7.1%)	4 (2.8%)	0.206	7 (4.2%)	11 (6.6%)	9 (5.5%)	0.626
CHF	No. (%)	12 (8.2%)	9 (6.4%)	6 (4.2%)	0.370	10 (6.0%)	14 (8.4%)	8 (4.9%)	0.413
CVA	No. (%)	12 (8.2%)	6 (4.3%)	5 (3.5%)	0.161	13 (7.8%)	11 (6.6%)	8 (4.9%)	0.556
CCI	No. (%)	4.6 (2.7)	4.5 (2.7)	3.9 (2.2)	0.069	4.4 (2.9)	4.6 (2.5)	4.3 (2.5)	0.457
Metformin use	No. (%)	16 (10.9%)	21 (15.0%)	28 (19.6%)	0.124	14 (8.4%)	27 (16.3%)	36 (22.1%)	0.003
Statin use	No. (%)	13 (8.9%)	18 (12.9%)	24 (16.8%)	0.134	11 (6.6%)	20 (12.1%)	26 (15.9%)	0.029
CCB use	No. (%)	32 (21.9%)	23 (16.4%)	36 (25.2%)	0.192	35 (21.1%)	37 (22.3%)	43 (26.4%)	0.492

*AFB, acid-fast bacilli; AMI, acute myocardial infarction; BMI, body mass index; CAD, coronary artery disease; CCB, calcium channel blocker; CCI, charlson comorbidity index; CHF, congestive heart failure; CKD, chronic kidney disease; COPD, chronic obstructive pulmonary disease; CVA, cerebrovascular accident; DM, diabetes mellitus; HIV, human immunodeficiency virus; HTN, hypertension; IQR, interquartile range; PVD, peripheral vascular disease; SD, standard deviation; TC, total cholesterol; TG, triglycerides*.

**Figure 1:**
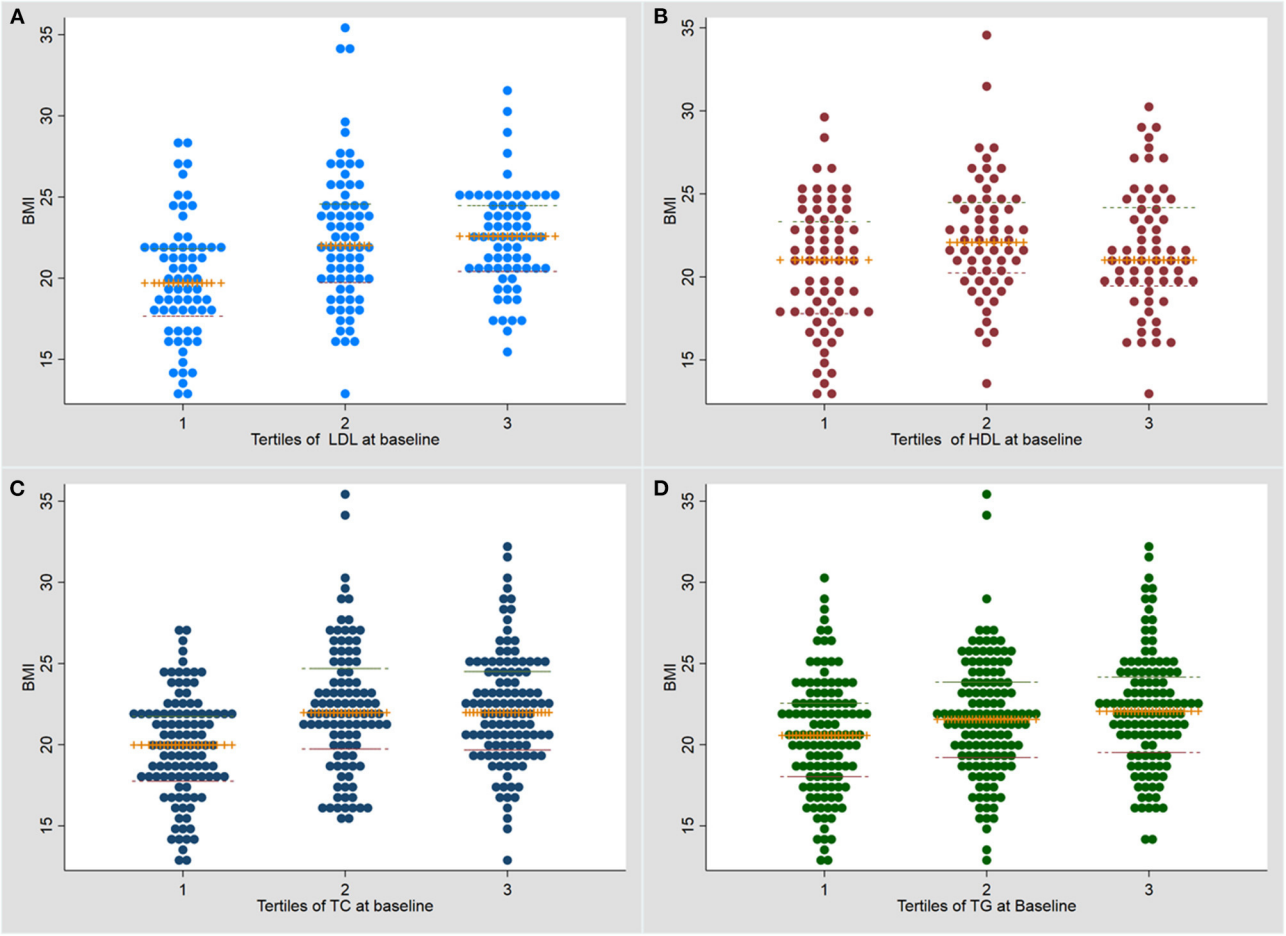
Dot plot for the association of baseline serum lipid levels with body mass index at baseline. **(A)** LDL (low density lipoprotein); **(B)** HDL (high density lipoprotein); **(C)** TC (total cholesterol); **(D)** Triglycerides (TG). TB, tuberculosis; BMI, body mass index.

BMI was significantly higher in the higher tertiles for each lipid (Figure 1). The association of tertiles of lipid levels with BMI using linear regression analysis is shown in Supplementary Table 1.

### Outcomes

Among 514 patients, 129 (26.6%) died due to any cause during a median follow up of 270 (IQR 206–270) days (Supplementary Table 2). A total of 72/514 patients (14.0%) died of infection-related causes, constituting 55.8% (72/129 patients) of all deaths during the first 9 months of TB treatment. Of this group, 4 patients (5.5%) died due to TB-related causes, 21 patients (29.2%) due to pneumonia and 45 patients (62.5%) due to sepsis. All-cause and infection-related mortality stratified by lipid levels are shown in Table 3.

**Table 3:** Association of serum lipid levels with all-cause and infection-related mortality by Cox regression analysis.

**Type of lipid**	**Total patient (N)**	**9-month all-cause mortality**							**9-month Infection related mortality**						
		***n* (%)**	**Unadjusted HR**	**(95%CI)**	***p*-value**	**Adjusted HR^**#**^**	**95%CI**	***p*-value**	***n* (%)**	**Unadjusted HR**	**(95%CI)**	***p*-value**	**Adjusted HR^**#**^**	**95%CI**	***p*-value**
LDL tertiles 1st	92	32 (37.2%)	Ref	–	–	Ref	–	–	19 (22.1%)	Ref	–	–	Ref	–	–
2nd	90	21 (24.1%)	0.64	0.37–1.17	0.117	1.03	0.50–2.12	0.933	13 (14.9%)	0.68	0.34–1.38	0.284	1.36	0.54–3.41	0.510
3rd	88	13 (19.7%)	0.38	0.19–0.72	0.003	0.60	0.24–1.49	0.273	4 (4.7%)	0.19	0.07–0.59	0.003	0.42	0.11–1.67	0.218
HDL tertiles 1st	83	32 (37.7%)	Ref	–	–	Ref	–	–	20 (23.5%)	Ref	–	–	Ref	–	–
2nd	86	21 (27.6%)	0.71	0.41–1.23	0.230	0.40	0.19–0.85	0.017	12 (15.8%)	0.66	0.32–1.35	0.259	0.43	0.16–1.19	0.105
3rd	80	9 (11.7%)	0.27	0.13–0.57	0.001	0.17	0.07–0.44	<0.001	3 (3.9%)	0.15	0.04–0.50	0.002	0.13	0.03–0.53	0.004
TC tertiles 1st	146	52 (38.8%)	Ref	–	–	Ref	–	–	34 (25.4%)	Ref	–	–	Ref	–	–
2nd	140	25 (18.4%)	0.44	0.28–0.72	0.001	0.54	0.29–0.99	0.049	11 (8.1%)	0.31	0.16–0.61	0.001	0.35	0.15–0.86	0.021
3rd	143	12 (8.8%)	0.19	0.11–0.37	<0.001	0.30	0.14–0.65	0.003	6 (4.4%)	0.16	0.07–0.37	<0.001	0.26	0.09–0.72	0.009
TG tertiles 1st	166	58 (38.2%)	Ref	–	–	Ref	–	–	30 (19.7%)	Ref	–	–	Ref	–	–
2nd	166	30 (18.9%)	0.47	0.30–0.73	0.001	0.58	0.33–1.01	0.054	18 (11.3%)	0.56	0.31–1.01	0.054	0.71	0.35–1.41	0.322
3rd	163	39 (25.0%)	0.65	0.43–0.97	0.037	1.08	0.65–1.81	0.765	23 (14.7%)	0.75	0.44–1.30	0.311	1.14	0.58–2.24	0.711

*LDL, low density lipoprotein cholesterol; HDL, high density lipoprotein cholesterol; TC, total cholesterol; TG, triglycerides; HR, hazard ratio*.

# *Adjusted for age; sex; BMI, body mass index; CCI, charlson comorbidity index; transplantation; alcoholism; smoking; initial AFB smear; presence of cavitary disease; metformin, statin, and calcium channel blocker use*.

*Ref, Reference group for the cox-regression analysis among the three tertiles*.

### Effect of Serum Lipid Levels on 9-Month All-Cause Mortality and Infection-Related Mortality

**Figure 2:**
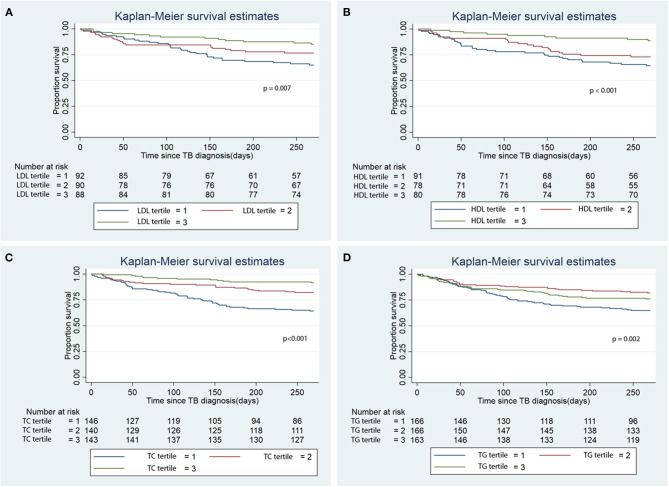
Kaplan Meier analysis of the association of baseline serum lipid level tertile with all-cause mortality. **(A)** Low density lipoprotein (LDL); **(B)** High density lipoprotein (HDL); **(C)** Total cholesterol (TC); **(D)** Triglycerides (TG). TB, tuberculosis.

During the first 9 months of ATT initiation, there was a lower hazard of all-cause mortality in univariable Cox regression for the 3rd tertiles of LDL (HR 0.38, 95% CI 0.19–0.72), HDL (HR 0.27, 95% CI 0.13–0.57), TC (HR 0.19, 95% CI 0.11–0.37) and TG (HR 0.65, 0.43–0.97) (Table 3). The log-rank test of the Kaplan-Meier analysis showed that patients classified in the 3rd tertiles of LDL, HDL, TC and TG had significantly longer survival compared to the other tertiles (Figure 2; *p* < 0.001). After adjusting for confounders, namely age, sex, BMI, CCI, transplantation, alcohol use disorder, smoking, initial AFB smear positivity, presence of cavitations, metformin, statin, and calcium channel blocker use for each of the serum lipid levels, lower hazards of all-cause mortality were only noted in the 2nd and 3rd tertiles of HDL and TC compared to the 1st tertile (Table 3). Serum LDL and TG did not a show significant association with all-cause mortality in the multivariable analysis.

**Figure 3:**
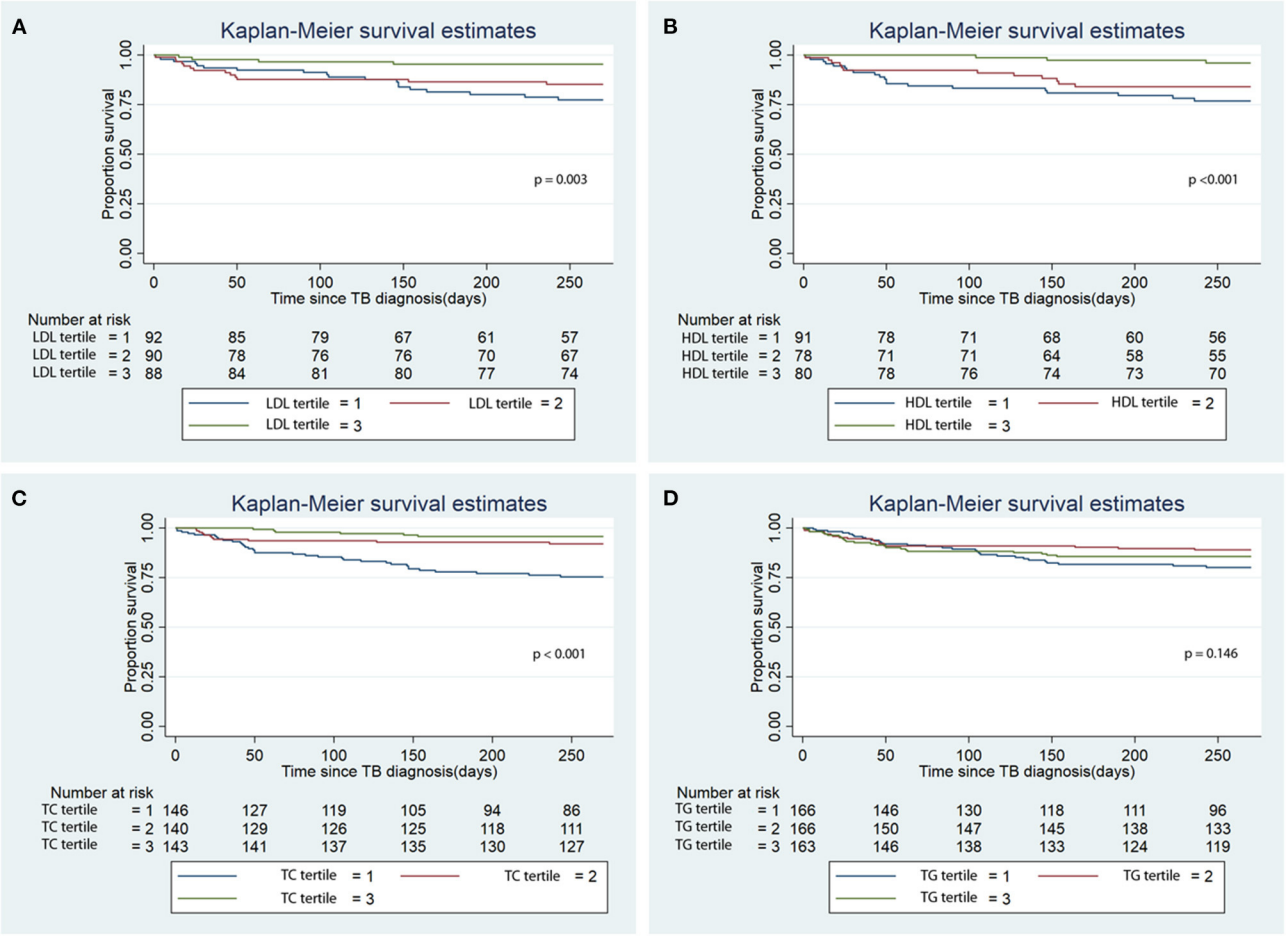
Kaplan Meier analysis of the association of baseline serum lipid level tertiles with infection-related mortality. **(A)** Low density lipoprotein (LDL); **(B)** High density lipoprotein (HDL); **(C)** Total cholesterol (TC); **(D)** Triglycerides (TG). TB, tuberculosis.

There was lower infection-related mortality in the univariable Cox regression analysis in the 3rd tertile of HDL and 2nd and 3rd tertiles of LDL and TC compared to the 1st tertiles of the corresponding lipids (Table 3). Kaplan-Meier analysis showed that patients in the 3rd tertiles of LDL, HDL, and TC significantly longer survival from infection-related mortality compared to the 1st and 2nd tertiles (Figure 3; *p* < 0.001). After adjusting for confounders, lower hazards of infection-related mortality were noted in the 3rd tertile of HDL and the 2nd and 3rd tertiles of TC (Table 3). Serum LDL and TG showed no association with infection-related mortality in the multivariable analysis.

#### Sensitivity Analysis

Sensitivity analysis after excluding patients who died within the first month of ATT initiation showed a similar lower all-cause and infection-related mortality for the 3rd tertiles of HDL and TC but not LDL (Supplementary Table 3). The 2nd tertile of TG showed significantly lower all-cause mortality compared to the 1st and 3rd tertiles. A similar association was noted for infection-related mortality but was non-significant. Similar findings were noted during sensitivity analysis using mean lipid levels during TB treatment (Supplementary Table 4).

### Effect of Serum Lipid Levels on Baseline Inflammatory Markers

**Figure 4:**
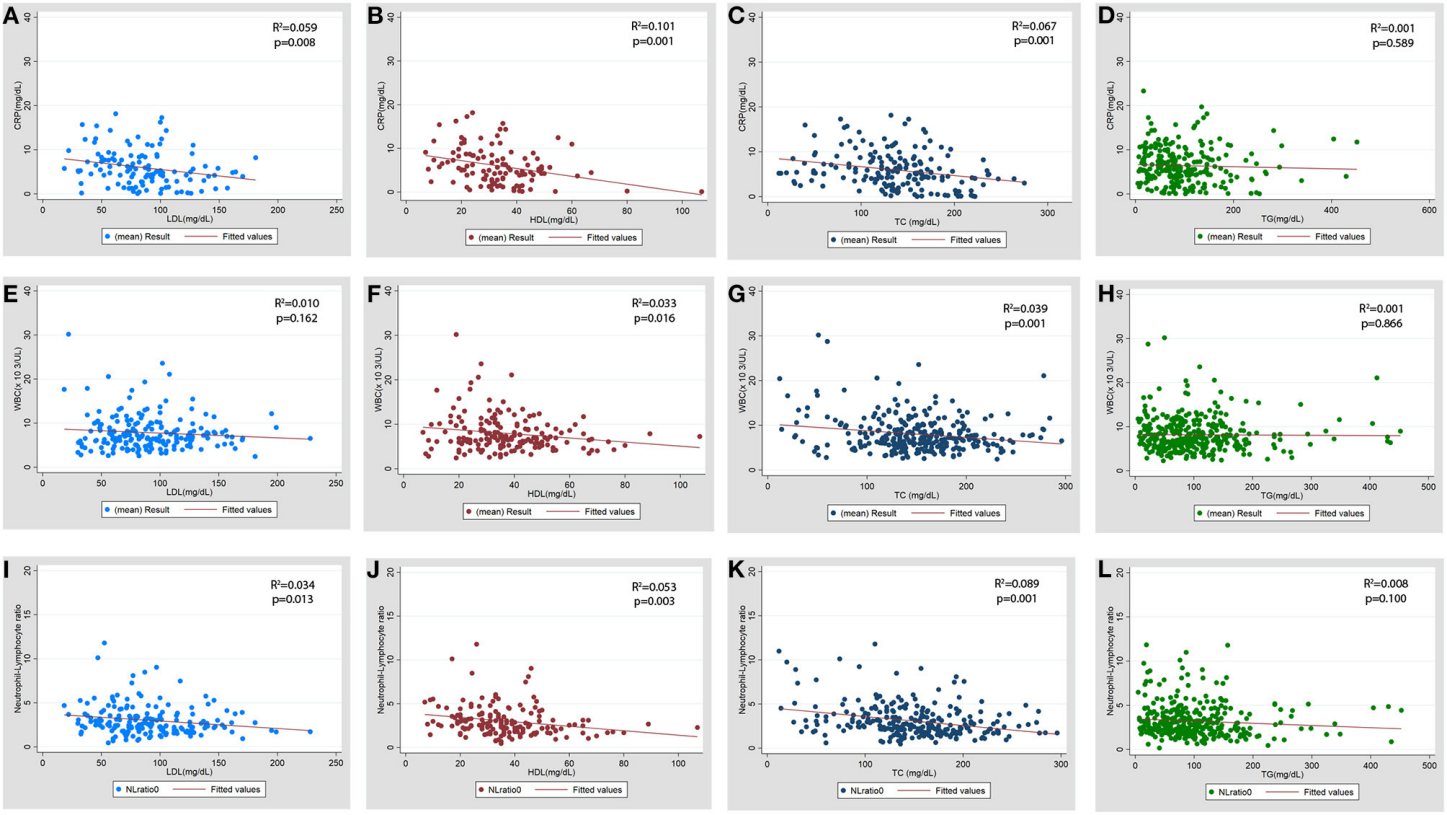
Scatter plot for the association of baseline serum lipid levels with baseline inflammatory markers. **(A–D)** CRP (C-reactive protein), **(E–H)** WBC (total leukocyte count x10^3^/μL), **(I-L)** NL ratio (neutrophil lymphocyte ratio). LDL, low density lipoprotein; HDL, high density lipoprotein; TC, total cholesterol; TG, triglycerides.

The association of serum lipid levels with serum inflammatory markers are shown in Table 4 and Figure 4. The 3rd tertiles of HDL and TC were associated with significantly lower levels of CRP, WBC, and NL ratio, while the 3rd tertile of LDL and 2nd tertile of TG were associated only with lower CRP levels in the multivariable model (Table 4). When lipid levels were considered as continuous variables, each 10 mg/dL increase in baseline serum HDL and TC was associated with lower CRP levels by 0.79 mg/dL (*p* = 0.007) and 0.15 mg/dL (*p* = 0.026), respectively, in multivariable linear regression analysis (Supplementary Table 5). The analysis for ESR was not performed because of a very low proportion of patients (2.3%) with these test results. Multivariable linear regression models showed a consistent inverse relationship between elevated HDL and TC levels and WBC and NL ratio (Table 4). LDL levels were not associated with lower WBC or NL ratios in the multivariable models. The 2nd tertile of TG levels showed lower levels of CRP compared to the 1st tertile. However, there was no association between TG levels and WBC, or NL ratio.

**Table 4:** Association of serum lipid level tertiles with CRP, TLC and NL ratio using linear regression analysis.

**Type of lipid**	**CRP (mg/dL)**					**Total Leukocyte count (x 10**^****3****^**/μL)**					**NL ratio**				
	**Mean (SD)**	**Univariable B (SE)**	***p*-value**	**Multivariable B (SE)^**#**^**	***p*-value**	**Mean (SD)**	**Univariable B (SE)**	***p*-value**	**Multivariable B (SE)^**#**^**	***p*-value**	**Mean (SD)**	**Univariable B (SE)**	***p*-value**	**Multivariable B (SE)^**#**^**	***p*-value**
LDL tertiles 1st	7.3 (4.2)	Ref	–	Ref	–	8.0 (4.6)	Ref	–	Ref	–	3.3 (1.9)	Ref	–	Ref	–
2nd	5.9 (4.1)	−1.36 (0.85)	0.113	−1.71 (1.14)	0.138	8.1 (4.1)	0.13 (0.71)	0.857	0.38 (0.80)	0.634	3.2 (1.8)	−0.11 (0.31)	0.720	0.06 (0.40)	0.16
3rd	4.1 (3.4)	−3.11 (0.98)	0.002	−2.54 (1.27)	0.049	7.4 (3.1)	−0.66 (0.69)	0.336	−1.18 (0.78)	0.126	2.5 (1.2)	−0.75 (0.31)	0.015	−0.65 (0.39)	0.098
HDL tertiles 1st	7.4 (4.3)	Ref	–	Ref	–	8.9 (5.4)	Ref	–	Ref	–	3.5 (1.9)	Ref	–	Ref	–
2nd	5.8 (4.4)	−1.83 (0.91)	0.047	−1.28 (1.17)	0.279	8.1 (3.6)	−0.81 (0.75)	0.284	−0.81 (0.81)	0.920	2.7 (1.4)	−0.79 (0.33)	0.018	−0.70 (0.42)	0.095
3rd	4.3 (3.1)	−3.12 (0.95)	0.001	−3.27 (1.15)	0.006	7.0 (2.4)	−1.86 (0.73)	0.011	−1.68 (0.74)	0.025	2.8 (1.7)	−0.76 (0.32)	0.019	−1.03 (0.39)	0.010
TC tertiles 1st	6.9 (4.1)	Ref	–	Ref	–	8.9 (4.9)	Ref	–	Ref	–	3.7 (2.2)	Ref	–	Ref	–
2nd	5.9 (4.0)	−0.87 (0.68)	0.201	−0.53 (0.87)	0.545	7.8 (3.5)	−1.15 (0.56)	0.041	−0.11 (0.56)	0.846	2.8 (1.5)	−0.88 (0.27)	0.001	−0.50 (0.29)	0.093
3rd	3.9 (3.4)	−2.89 (0.78)	<0.001	−2.73 (0.96)	0.005	7.2 (2.9)	−1.69 (0.56)	0.003	−1.37 (0.57)	0.017	2.6 (1.5)	−1.05 (0.26)	<0.001	−0.90 (0.29)	0.002
TG tertiles 1st	7.1 (4.1)	Ref	–	Ref	–	8.3 (4.2)	Ref	–	Ref	–	3.4 (1.9)	Ref	–	Ref	–
2nd	5.2 (3.4)	−1.93 (0.65)	0.003	−1.84 (0.82)	0.026	7.9 (3.6)	−0.38 (0.49)	0.437	−0.05 (0.53)	0.922	3.1 (2.0)	−0.35 (0.26)	0.178	−0.50 (0.29)	0.090
3rd	6.8 (4.7)	−0.35 (0.66)	0.596	0.39 (0.84)	0.634	8.3 (3.7)	−0.06 (0.51)	0.902	0.28 (0.57)	0.624	3.1 (1.8)	−0.34 (0.26)	0.197	−0.46 (0.32)	0.157

*LDL, Low density lipoprotein cholesterol; HDL, High density lipoprotein cholesterol; TC, Total cholesterol; TG, Triglycerides; B, Linear regression co-efficient; SE, standard error; CRP, C-reactive protein; NL ratio, neutrophil-lymphocyte ratio*.

# *Adjusted for age; sex; BMI, body mass index; CCI, charlson comorbidity index; transplantation; alcoholism; smoking; initial AFB smear; presence of cavitary disease; metformin, statin, and calcium channel blocker use*.

*Ref, Reference group for the cox-regression analysis among the three tertiles*.

#### Sensitivity Analysis

We performed sensitivity analyses by restricting the baseline lipid levels and inflammatory markers to the first 15 days of TB treatment and found that the 3rd tertiles of HDL were associated with lower CRP and NL ratio, while the 3rd tertile of TC was associated with lower CRP, WBC, and NL ratio in the multivariable model. The 3rd tertile of LDL and the 2nd tertile of TG were associated with non-significantly lower inflammatory markers compared to the 1st tertile in the multivariable model (Supplementary Table 6).

### Test of Effect Modification by BMI on the Association of Lipid Levels With Mortality and the Level of Inflammation

Stratified analysis based on three different cut-offs of BMI (20, 21, and 22 Kg/m^2^) were performed. The strata with high lipid levels (3rd tertile) and BMI greater than the cut-offs (20, 21, and 22 Kg/m^2^) showed significantly lower hazard of all-cause and infection-related mortality and lower odds of elevated inflammatory markers, namely CRP > 5 mg/dL, WBC >10 X 10^3^/μL, and NL ratio >4 (Supplementary Tables 7–11). We found no evidence of effect modification for the lipid levels by BMI for the above outcomes across any of the cut offs of BMI (Supplementary Tables 7–11).

## Discussion

In our retrospective cohort, we found that higher serum cholesterol levels were associated with lower hazards of all-cause and infection-related mortality during ATT, independent of confounding factors. While higher serum LDL showed lower hazard ratios for mortality in univariable Cox regression alone, higher serum HDL and TC showed this association in both univariable and multivariable analyses. The linear regression model demonstrated that higher levels of LDL, HDL and TC were independently associated with lower levels of inflammatory markers such as CRP, WBC, and NL ratio.

Preclinical studies have found that *Mtb* can manipulate host lipids in several ways. *Mtb* induces host lipid accumulation, primarily TG and cholesterol esters, to promote its intra-macrophage survival, leading to the formation of lipid-laden foam cells (19, 20). In addition to serving as a nutrient reservoir for dormant *Mtb* (21), these host lipids impair the ability of macrophages to kill intracellular bacilli.

Prior literature has shown that active TB patients have decreased serum cholesterol levels (6–8). Lower serum levels of LDL, HDL and TC correlated with more extensive TB lung disease (7, 8), such as larger caseous lesions and severe fibro-encapsulation (22). Consistent with these data, lower serum levels of cholesterol have been associated with increased proportion of sputum smear positivity for *Mtb* (7, 22). Conversely, adult TB patients receiving a cholesterol-rich diet (800 mg/d cholesterol) experienced accelerated sterilization of sputum *Mtb* cultures during ATT relative to controls receiving a normal diet (250 mg/d cholesterol) (23). In our study, we found that the highest tertiles of serum LDL, HDL and TC levels were associated with lower hazards of mortality in the first 9 months of ATT.

CRP and NL ratio are important markers of systemic inflammation in TB (24). Though correlation is demonstrated between serum cholesterol levels and systemic inflammatory markers in TB, an association after adjusting for other confounders is not established (7). We showed that among active TB patients, higher serum HDL and TC are independently associated with lower CRP, WBC, and NL ratio in both univariable and multivariable linear regression (Table 4).

The reason for the association of higher serum cholesterol with lower systemic inflammation is currently unknown. Murine models have shown that hypercholesterolemia results in a hyperinflammatory response to *Mtb* infection (25) and impairs host defenses against TB (26). It has been hypothesized that lower serum lipids during inflammation may be due to an acute phase response (27). Preclinical studies revealed, in response to inflammation, an increase in secretory phospholipase A2 and serum amyloid A, thereby decreasing HDL levels (28, 29). Additionally, reverse cholesterol transport mediated by ATP-binding cassette transporter (ABC) and lecithin-cholesterol acyltransferase (LCAT) are decreased during inflammation, leading to decreased HDL (30). Lower serum lipid levels are also associated with greater levels of systemic inflammation in other infections (9–11, 31–33). A similar clinical scenario has been explored in patients with rheumatoid arthritis (RA). Patients with RA had a more atherogenic lipid profile (high LDL and TC, low HDL) prior to symptom development (34), with a significant decline in lipid levels following RA development when compared to healthy controls (35). Furthermore, there was an increase in lipid levels following anti-inflammatory therapy with TNF-alpha inhibitors (36). No current evidence supports that low serum lipids predispose to greater inflammation. Thus, lower lipid levels are more likely a result, rather than a cause, of increased inflammation in TB disease.

TB patients have higher degrees of wasting, with relatively low serum levels of amino acids, cholesterol, fatty acids and phospholipid metabolites (37). Malnutrition, suggested by low BMI, is associated with an increased risk of TB (38), and with worse TB outcomes, such as mortality (13), poor sputum conversion rates (39), and greater radiographic severity (40). Additionally, higher BMI is correlated with improved survival and earlier sputum conversion (12, 13, 41). The available evidence regarding the association of BMI with the levels of inflammation in TB is unclear. Studies show that increased BMI is associated with increased (42), decreased (43) or unchanged (44) serum levels of pro-inflammatory cytokines. Though serum cholesterol and TG levels showed a direct relationship with BMI, beyond certain inflection points in BMI, the slope in the association with lipid levels decreased to a plateau. In our cohort, BMI did not significantly confound or modify the association of lipid levels with mortality or inflammation at various cut-off levels. Thus, we have demonstrated that the effect of lipid levels on the outcomes are independent of nutritional status.

Our study has several strengths. Our larger sample size with relevant data on the confounders enabled the inferences obtained in our study. Treatment options were standardized at the study center, thereby decreasing the heterogeneity in the treatment received. We note a few limitations, including the lack of standardized periods for follow-up data on serum lipid levels, which would have enabled an assessment of trend of lipids during the course of ATT. Availability of additional lab markers, like serum albumin, would have facilitated further assessment of patients' nutritional status. We were unable to evaluate the association of lipid levels with TB-specific outcomes, such as sputum conversion and lung function from this subset of our patient cohort. Ours is a single center study with a high proportion of elderly patients, and multicenter cohorts might help to corroborate our results.

## Conclusions

In summary, we demonstrated that higher serum cholesterol levels are associated with lower hazards of all-cause and infection-related mortality during ATT and lower levels of systemic inflammation at baseline, independent of BMI. There was no effect modification by BMI on the above associations. Cohort studies assessing TB incidence might enable us to determine whether lower lipid levels are the cause or the effect of severe TB disease. An improved understanding of this relationship might provide further insights into TB pathogenesis and help to risk-stratify patients based on serum lipid levels.

## Data Availability Statement

The raw data supporting the conclusions of this article will be made available by the authors, without undue reservation.

## Ethics Statement

The studies involving human participants were reviewed and approved by Johns Hopkins University and National Taiwan University Hospital. Written informed consent for participation was not required for this study in accordance with the national legislation and the institutional requirements.

## Author Contributions

VC and PK conceived the study. J-YW collected the data. VC performed the analysis. AG guided in the analysis and the methods. VC, LZ, JR, and AK drafted the manuscript. AG, J-YW, and PK corrected the draft. All authors contributed to the article and approved the submitted version.

## Conflict of Interest

The authors declare that the research was conducted in the absence of any commercial or financial relationships that could be construed as a potential conflict of interest.

## Abbreviations

TB: tuberculosis
MTB: mycobacterium tuberculosis (organism)
NTUH: National Taiwan University Hospital
LDL: low density lipoprotein cholesterol
HDL: high density lipoprotein cholesterol
TC: total cholesterol
TG: triglyceride
CRP: C-reactive protein
WBC: total leucocyte count
NL ratio: neutrophil lymphocyte ratio
ESR: erythrocyte sedimentation rate
ATT: TB treatment
BMI: body mass index
RA: rheumatoid arthritis
ABC: ATP- binding cassette protein
CKD: chronic kidney disease
RERI: relative excess of risk index
ANOVA: analysis of variance (Statistical test).
