# Supplementary material for: Higher Serum Cholesterol Levels Are Associated With Reduced Systemic Inflammation and Mortality During Tuberculosis Treatment Independent of Body Mass Index
Source: Front Cardiovasc Med. 2021 Jun 22;8:696517. doi: 10.3389/fcvm.2021.696517 (PMC8257940; doi:10.3389/fcvm.2021.696517)
Supplement: Supplementary file 1 [file Data_Sheet_1.PDF]

Supplementary Document

Supplementary Figure 1: Flow-diagram for patient selection

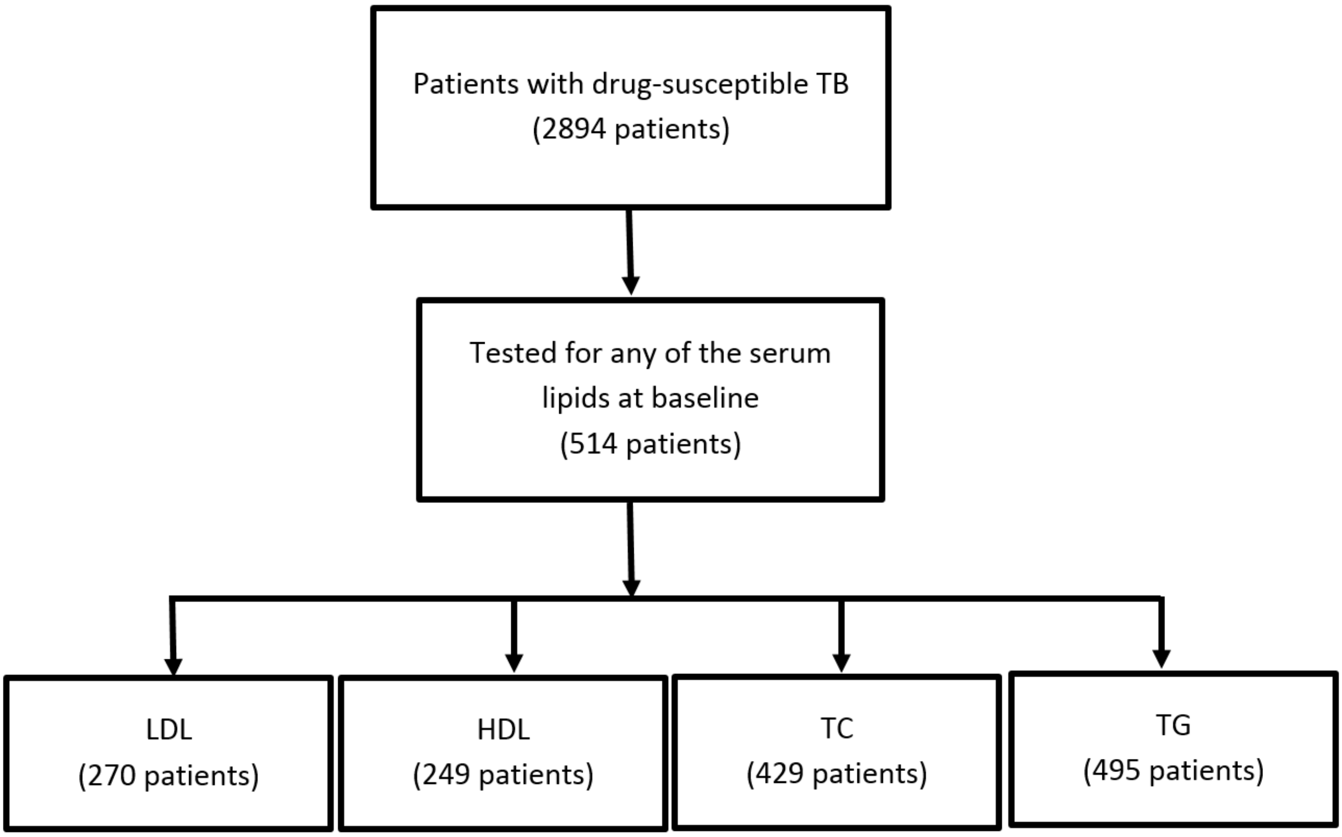

LDL, Low Density Lipoprotein Cholesterol; HDL, High Density Lipoprotein Cholesterol; TC, Total Cholesterol; TG, Triglycerides.

The numbers of patients mentioned below each of the Lipids (LDL, HDL, TC and TG) are the availability of the tests results and are overlapping.

Supplementary table 1: Value of lipid levels in each of the corresponding lipid tertiles and their association with BMI using linear regression analysis.

Type of Lipid	No. of patients	Lipid levels (mg/dL) Median (Range)	BMI (kg/m^2)		
			Mean (SD)	Univariable B (SE)	p-value[#]
LDL tertiles 1st	92	52.7 (6.2 – 73.4)	19.9 (3.7)	Ref	<0.001
2nd	90	87.8 (73.6 – 102)	22.5 (4.3)	2.56 (0.63)	
3rd	88	131.6 (102.4 -228)	22.4 (3.1)	2.51 (0.64)	
HDL tertiles 1st	83	21.0 (4-29)	20.6 (3.8)	Ref	0.024
2nd	86	34.3 (30-40)	22.4 (3.6)	1.78 (0.65)	
3rd	80	52.1 (41-107)	21.6 (3.7)	1.02 (0.65)	
TC tertiles 1st	146	98.6 (12-133)	19.9 (3.2)	Ref	< 0.001
2nd	140	152.2 (134-173)	22.3 (3.9)	2.38 (0.49)	
3rd	143	206.9 (173.5-320)	22.2 (3.5)	2.29 (0.47)	
TG tertiles 1st	166	45.2 (5-71)	20.5 (3.5)	Ref	0.002
2nd	166	92.1 (71.5-119)	21.6 (3.6)	1.05 (0.45)	
3rd	163	192.2(120-558.8)	22.1 (3.6)	1.57 (0.45)	

LDL, Low Density Lipoprotein Cholesterol; HDL, High Density Lipoprotein Cholesterol; TC, Total Cholesterol; TG, Triglycerides; B, linear regression co-efficient; SE – Standard error

[#] p-values obtained in this table are through univariable linear regression analysis

Supplementary Table 2: All-cause and infection-related mortality according to serum lipid level tertiles

Characteristic	No. of patients	Lipid levels (mg/dL) Median (Range)	9-month All-cause Mortality		9-month infection related mortality	
			N (%)	p-value[#]	N (%)	p-value[#]
Total	514	-	129 (26.6%)	-	72 (14.0%)	-
LDL tertiles 1st	92	52.7 (6.2 – 73.4)	32(37.2%)	0.004	19(22.1%)	0.004
2nd	90	87.8 (73.6 – 102)	21(24.1%)		13(14.9%)	
3rd	88	131.6 (102.4 -228)	13(19.7%)		4(4.7%)	
HDL tertiles 1st	83	21.0 (4-29)	32(37.7%)	0.001	20(23.5%)	0.002
2nd	86	34.3 (30-40)	21(27.6%)		12(15.8%)	
3rd	80	52.1 (41-107)	9(11.7%)		3(3.9%)	
TC tertiles 1st	146	98.6 (12-133)	52(38.8%)	<0.001	34(25.4%)	<0.001
2nd	140	152.2 (134-173)	25(18.4%)		11(8.1%)	
3rd	143	206.9 (173.5-320)	12(8.8%)		6(4.4%)	
TG tertiles 1st	166	45.2 (5-71)	58(38.2%)	0.001	30(19.7%)	0.116
2nd	166	92.1 (71.5-119)	30(18.9%)		18(11.3%)	
3rd	163	192.2(120-558.8)	39(25.0%)		23(14.7%)	

LDL, Low density lipoprotein cholesterol; HDL, High density Llpoprotein cholesterol; TC, Total cholesterol; TG, Triglycerides.

[#] p-values in the table are obtained from chi-square tests.

Supplementary table 3: Sensitivity analysis to assess the association between lipid levels with all-cause and infection related mortality by Cox regression.

(Excluded patients who died within the first month of TB treatment)

Characteristic	9-month All-cause Mortality						9-month Infection related mortality					
	Unadjusted HR	(95%CI)	p-value	Adjusted HR[#]	95%CI	p-value	Unadjusted HR	(95%CI)	p-value	Adjusted HR[#]	95%CI	p-value
LDL tertiles 1st	Ref	-	-	Ref	-	-	Ref	-	-	Ref	-	-
2nd	0.52	0.27-0.99	0.048	0.77	0.32-1.87	0.569	0.45	0.17-1.17	0.101	0.91	0.26-3.23	0.887
3rd	0.34	0.17-0.71	0.004	0.51	0.18-1.46	0.210	0.14	0.03-0.61	0.009	0.40	0.07-2.23	0.297
HDL tertiles 1st	Ref	-	-	Ref	-	-	Ref	-	-	Ref	-	-
2nd	0.67	0.35-1.27	0.217	0.35	0.14-0.91	0.032	0.53	0.20-1.42	0.209	0.19	0.03-1.14	0.069
3rd	0.31	0.14-0.69	0.004	0.15	0.05-0.45	0.001	0.23	0.07-0.82	0.023	0.20	0.05-0.89	0.034
TC tertiles 1st	Ref	-	-	Ref	-	-	Ref	-	-	Ref	-	-
2nd	0.36	0.21-0.64	<0.001	0.34	0.16-0.75	0.007	0.11	0.03-0.36	<0.001	0.05	0.01-0.42	0.006
3rd	0.22	0.11-0.42	<0.001	0.28	0.12-0.64	0.003	0.20	0.08-0.49	<0.001	0.29	0.09-0.86	0.026
TG tertiles 1st	Ref	-	-	Ref	-	-	Ref	-	-	Ref	-	-
2nd	0.38	0.23-0.63	<0.001	0.46	0.24-0.87	0.018	0.34	0.16-0.73	0.006	0.49	0.19-1.21	0.121
3rd	0.52	0.33-0.83	0.007	0.89	0.48-1.68	0.732	0.48	0.24-0.96	0.037	0.64	0.26-1.59	0.335

LDL, Low Density Lipoprotein Cholesterol; HDL, High Density Lipoprotein Cholesterol; TC, Total Cholesterol; TG, Triglycerides; HR, Hazard ratio.

#adjusted for Age; sex; BMI- Body mass index; CCI- Charlson Comorbidity Index; Transplantation; Alcoholism; smoking; initial AFB smear; presence of cavitary disease; metformin, statin, and calcium channel blocker use

Ref = Reference group for the cox-regression analysis among the three tertiles

Supplementary table 4: Sensitivity analysis for the association of mean serum lipid levels during TB treatment with all-cause and infection-related mortality by Cox regression.

Characteristic	9-month All-cause Mortality						9-month Infection related mortality					
	Unadjusted HR	(95%CI)	p-value	Adjusted HR[#]	95%CI	p-value	Unadjusted HR	(95%CI)	p-value	Adjusted HR[#]	95%CI	p-value
LDL tertiles 1st	Ref	-	-	Ref	-	-	Ref	-	-	Ref	-	-
2nd	0.76	0.38-1.53	0.441	1.18	0.43-3.24	0.743	0.83	0.36-1.91	0.658	1.15	0.35-3.81	0.819
3rd	0.53	0.25-1.16	0.112	0.94	0.29-3.08	0.923	0.25	0.07-0.87	0.030	0.38	0.07-2.13	0.271
HDL tertiles 1st	Ref	-	-	Ref	-	-	Ref	-	-	Ref	-	-
2nd	0.49	0.28-0.86	0.013	0.33	0.16-0.70	0.004	0.51	0.25-1.05	0.067	0.33	0.12-0.89	0.029
3rd	0.22	0.10-0.45	<0.001	0.11	0.04-0.31	<0.001	0.08	0.02-0.34	0.001	0.07	0.01-0.35	0.001
TC tertiles 1st	Ref	-	-	Ref	-	-	Ref	-	-	Ref	-	-
2nd	0.45	0.28-0.71	0.001	0.53	0.29-0.97	0.039	0.29	0.15-0.57	<0.001	0.36	0.16-0.83	0.016
3rd	0.18	0.09-0.34	<0.001	0.25	0.11-0.55	0.001	0.12	0.05-0.31	<0.001	0.19	0.06-0.57	0.003
TG tertiles 1st	Ref	-	-	Ref	-	-	Ref	-	-	Ref	-	-
2nd	0.47	0.30-0.73	0.001	0.58	0.33-1.02	0.058	0.59	0.33-1.06	0.077	0.72	0.36-1.46	0.366
3rd	0.64	0.42-0.95	0.028	1.04	0.61-1.76	0.896	0.71	0.41-1.23	0.217	0.92	0.45-1.87	0.811

LDL, Low Density Lipoprotein Cholesterol; HDL, High Density Lipoprotein Cholesterol; TC, Total Cholesterol; TG, Triglycerides; HR, Hazard ratio.

#adjusted for Age; sex; BMI- Body mass index; CCI- Charlson Comorbidity Index; Transplantation; Alcoholism; smoking; initial AFB smear; presence of cavitary disease; metformin, statin and calcium channel blocker use

Ref = Reference group for the cox-regression analysis among the three tertiles

Supplementary Table 5: Association of serum lipid levels with CRP, TLC and NL ratio using linear regression analysis:

Characteristic	CRP (mg/dL)				Total Leukocyte count (x 10^3/µL)				NL ratio			
	Univariable **B (SE)**	**p-value**	Multivariable **B (SE)#**	**p-value**	Univariable **B (SE)**	**p-value**	Multivariable **B (SE)#**	**p-value**	Univariable **B (SE)**	**p-value**	Multivariable **B (SE)#**	**p-value**
LDL (10mg/dl)	-0.29 (0.11)	0.008	-0.17 (0.13)	0.189	-0.1 (0.1)	0.162	-0.06 (0.08)	0.423	-0.08 (0.03)	0.013	-0.06 (0.04)	0.148
HDL (10mg/dl)	-0.89 (0.26)	0.001	-0.79 (0.29)	0.007	-0.5 (0.2)	0.017	-0.4 (0.2)	0.024	-0.25 (0.08)	0.003	-0.24 (0.09)	0.011
TC (10mg/dl)	-0.19 (0.06)	0.001	-0.15 (0.07)	0.026	-0.2 (0.04)	0.001	-0.1 (0.04)	0.003	-0.10 (0.02)	<0.001	-0.09 (0.02)	<0.001
TG (10mg/dl)	-0.02 (0.04)	0.532	0.0 (0.04)	0.992	-0.01 (0.03)	0.844	-0.01 (0.03)	0.645	-0.02 (0.01)	0.097	-0.02 (0.02)	0.278

LDL, Low Density Lipoprotein Cholesterol; HDL, High Density Lipoprotein Cholesterol; TC, Total Cholesterol; TG, Triglycerides; B, linear regression co-efficient; SE – Standard error; CRP, C-reactive protein; NL ratio, neutrophil-lymphocyte ratio.

#adjusted for Age; sex; BMI- Body mass index; CCI- Charlson Comorbidity Index; Transplantation; Alcoholism; smoking; initial AFB smear; presence of cavitary disease; metformin, statin, and calcium channel blocker use

Supplementary Table 6: Sensitivity analysis - Association of tertiles of serum lipid levels with CRP, TLC and NL ratio using linear regression analysis.
(Only including values of lipid levels and inflammatory markers performed within the first 15 days of TB diagnosis)

Characteristic	CRP (mg/dL)				Total Leukocyte count (x 10^3/µL)				NL ratio			
	Univariable **B (SE)**	**p-value**	Multivariable **B (SE)#**	**p-value**	Univariable **B (SE)**	**p-value**	Multivariable **B (SE)#**	**p-value**	Univariable **B (SE)**	**p-value**	Multivariable **B (SE)#**	**p-value**
LDL tertiles 1st	Ref	-	Ref	-	Ref	-	Ref	-	Ref	-	Ref	-
2nd	-1.57 (1.30)	0.234	-2.27 (1.79)	0.214	0.23 (0.83)	0.779	0.1 (0.88)	0.882	-0.16 (0.38)	0.673	-0.06 (0.44)	0.891
3rd	-3.94 (1.46)	0.010	-3.65 (1.88)	0.061	-0.38 (0.82)	0.647	-0.74 (0.86)	0.394	-0.54 (0.39)	0.169	-0.23 (0.44)	0.605
HDL tertiles 1st	Ref	-	Ref	-	Ref	-	Ref	-	Ref	-	Ref	-
2nd	-1.45 (1.07)	0.181	-0.72 (1.25)	0.568	0.17 (0.81)	0.809	-0.05 (0.83)	0.952	-0.75 (0.33)	0.026	-0.75 (0.38)	0.049
3rd	-3.56 (1.14)	0.003	-3.34 (1.33)	0.014	-1.07 (0.15)	0.147	-1.06 (0.81)	0.188	-1.14 (0.33)	0.001	-1.07 (0.37)	0.004
TC tertiles 1st	Ref	-	Ref	-	Ref	-	Ref	-	Ref	-	Ref	-
2nd	-0.96 (0.82)	0.240	-1.03 (0.94)	0.276	-1.01 (0.59)	0.091	-0.63 (0.67)	0.353	-0.83 (0.26)	0.002	-0.66 (0.31)	0.034
3rd	-2.84 (0.92)	0.002	-2.82 (1.01)	0.006	-1.54 (0.59)	0.010	-1.71 (0.67)	0.011	-1.03 (0.26)	< 0.001	-1.00 (0.30)	0.001
TG tertiles 1st	Ref	-	Ref	-	Ref	-	Ref	-	Ref	-	Ref	-
2nd	-1.85 (0.76)	0.016	-1.31 (0.88)	0.139	-0.33 (0.53)	0.539	-0.42 (0.62)	0.500	-0.33 (0.26)	0.210	-0.36 (0.30)	0.236
3rd	0.18 (0.49)	0.818	1.00 (0.91)	0.271	-0.41 (0.54)	0.448	-0.43 (0.65)	0.514	-0.37 (0.27)	0.167	-0.39 (0.32)	0.217

LDL, Low Density Lipoprotein Cholesterol; HDL, High Density Lipoprotein Cholesterol; TC, Total Cholesterol; TG, Triglycerides; B, linear regression co-efficient; SE – Standard error; CRP, C-reactive protein; NL ratio, neutrophil-lymphocyte ratio.
#adjusted for Age; sex; BMI- Body mass index; CCI- Charlson Comorbidity Index; Transplantation; Alcoholism; smoking; initial AFB smear; presence of cavitary disease; metformin, statin, and calcium channel blocker use

Ref = Reference group for the cox-regression analysis among the three tertiles

Supplementary Table 7: Test of Effect modification of lipid levels on mortality by BMI with a cut-off value of 20kg/m2

Type of lipid	BMI cut-off	Mortality						HR (95%CI) # for mortality within each BMI stratum for high lipid (+) compared to (-)	RERI
		High lipid (-)			High lipid (+)				
		n/N	HR (95%CI) #	P-value	n/N	HR (95%CI) #	p-value		
All-cause mortality									
LDL	BMI<20<br>BMI>20	15/57<br>19/79	Ref<br>0.92 (0.47to1.81)	-<br>0.810	2/13<br>6/56	0.55 (0.13 to 2.41)<br>0.39 (0.15 to 1.01)	0.429<br>0.051	0.55 (0.13 to 2.41)<br>0.43 (0.17 to 1.07)	-0.08 (-1.15 to 0.99)<br>(p=0.882)
HDL	BMI<20<br>BMI>20	14/44<br>20/82	Ref<br>0.75 (0.38-1.49)	-<br>0.410	3/20<br>3/40	0.42 (0.12 to 1.44)<br>**0.20 (0.06 to 0.70)**	0.166<br>**0.012**	0.42 (0.12 to 1.44)<br>0.27 (0.08 to 0.92)	0.04 (-0.75 to 0.83)<br>(p=0.927)
TC	BMI<20<br>BMI>20	20/80<br>29/124	Ref<br>0.97 (0.55 to 1.71)	-<br>0.909	2/29<br>7/84	0.25 (0.06 to 1.08)<br>**0.31 (0.13 to 0.74)**	0.064<br>**0.008**	0.25 (0.06 to 1.07)<br>0.32 (0.14 to 0.74)	0.09 (-0.58 to 0.77)<br>(p=0.788)
TG	BMI<20<br>BMI>20	23/88<br>34/156	Ref<br>0.87 (0.51 to 1.47)	-<br>0.597	8/32<br>20/88	1.01 (0.45 to 2.26)<br>0.93 (0.51 to 1.69)	0.982<br>0.802	1.01 (0.45 to 2.26)<br>1.06 (0.61 to 1.85)	0.14 (-0.83 to 1.11)<br>(p=0.775)
Infection related mortality									
LDL	BMI<20<br>BMI>20	11/57<br>10/79	Ref<br>0.66 (0.28 to 1.55)	-<br>0.341	1/13<br>2/55	0.38 (0.05 to 2.95)<br>0.18 (0.04 to 0.81)	0.355<br>0.026	0.38 (0.05 to 2.95)<br>0.28 (0.06 to 1.27)	0.14 (-0.88 to 1.16)<br>(p=0.789)
HDL	BMI<20<br>BMI>20	10/44<br>10/82	Ref<br>0.53 (0.22 to 1.27)	-<br>0.156	2/20<br>1/40	0.39 (0.09 to 1.81)<br>**0.10 (0.01 to 0.75)**	0.232<br>**0.026**	0.39 (0.09 to 1.81)<br>0.19 (0.02 to 1.46)	0.17 (-0.67 to 1.01)<br>(p=0.692)
TC	BMI<20<br>BMI>20	15/80<br>15/124	Ref<br>0.67 (0.33 to 1.37)	-<br>0.271	1/29<br>4/84	**0.17 (0.02 to 1.30)**<br>**0.24 (0.08 to 0.73)**	**0.089**<br>**0.012**	0.17 (0.02 to 1.30)<br>0.37 (0.12 to 1.11)	0.40 (-0.21 to 1.02)<br>(p=0.199)
TG	BMI<20<br>BMI>20	15/88<br>20/156	Ref<br>0.78 (0.40 to 1.53)	-<br>0.472	6/32<br>10/88	1.15 (0.45 to 2.97)<br>0.71 (0.32 to 1.59)	0.764<br>0.407	1.15 (0.45 to 2.97)<br>0.91 (0.42 to 1.19)	-0.23 (-1.47 to 1.02)<br>(p=0.764)

High Lipid (+) = 3rd tertile: High Lipid (-) = 1st or 2nd tertile; RERI = relative excess risk due to interaction.
LDL, Low Density Lipoprotein Cholesterol; HDL, High Density Lipoprotein Cholesterol; TC, Total Cholesterol; TG, Triglycerides.
#adjusted for Age; sex; CCI- Charlson Comorbidity Index; Transplantation; Alcoholism; smoking; initial AFB smear; presence of cavitary disease; metformin, statin, and calcium channel blocker use
Ref = Reference group for the stratified analysis

Supplementary Table 8: Test of effect modification of lipid levels on mortality by BMI (cut-off value of 21kg/m^2) #

Type of lipid	BMI cut-off	Mortality						HR (95%CI) # for mortality within each BMI stratum for high lipid (+) compared to (-)	RERI
		High lipid (-)			High lipid (+)				
		n/N	HR (95%CI) #	P-value	n/N	HR (95%CI) #	p-value		
**All-cause mortality**									
LDL	BMI<21<br>BMI>21	17/67<br>17/69	Ref<br>1.01 (0.52 to1.97)	-<br>0.977	4/25<br>4/43	0.61 (0.20 to 1.80)<br>0.35 (0.12 to 1.04)	0.368<br>0.059	0.61 (0.20 to 1.80)<br>0.35 (0.12 to 1.05)	-0.26 (-1.29 to 0.76)<br>(p=0.613)
HDL	BMI<21<br>BMI>21	16/54<br>18/72	Ref<br>0.84 (0.43 to 1.65)	-<br>0.613	4/30<br>2/30	0.39 (0.13 to 1.16)<br>**0.19 (0.05 to 0.87)**	0.091<br>**0.032**	0.38 (0.13 to 1.13)<br>0.24 (0.06 to 1.04)	-0.03 (-0.82 to 0.76)<br>(p=0.943)
TC	BMI<21<br>BMI>21	23/94<br>26/110	Ref<br>1.03 (0.59 to 1.80)	-<br>0.925	4/47<br>5/66	**0.32 (0.11 to 0.95)**<br>**0.29 (0.11 to 0.77)**	**0.039**<br>**0.013**	**0.32 (0.11 to 0.95)**<br>**0.29 (0.11 to 0.75)**	-0.06 (-0.77 to 0.65)<br>(p=0.865)
TG	BMI<21<br>BMI>21	27/115<br>30/129	Ref<br>1.05 (0.62 to 1.7)	-<br>0.856	15/75<br>13/45	1.32 (0.68 to 2.55)<br>0.91 (0.48 to 1.71)	0.418<br>0.769	1.32 (0.68 to 2.55)<br>0.87 (0.47 to 1.61)	-0.45 (-1.53 to 0.63)<br>(p=0.409)
**Infection related mortality**									
LDL	BMI<21<br>BMI>21	11/67<br>10/69	Ref<br>0.92 (0.39 to 2.16)	-<br>0.843	2/25<br>1/43	0.47 (0.11 to 2.14)<br>0.14 (0.02 to 1.07)	0.331<br>0.057	0.47 (0.11 to 2.14)<br>0.70 (0.42 to 1.19)	-0.25 (-1.43 to 0.92)<br>(p=0.672)
HDL	BMI<21<br>BMI>21	11/54<br>9/72	Ref<br>0.62 (0.26 to 1.48)	-<br>0.280	2/30<br>1/30	0.29 (0.06 to 1.30)<br>0.15 (0.02 to 1.15)	0.106<br>0.068	0.29 (0.06 to 1.30)<br>0.25 (0.03 to 1.97)	0.25 (-0.53 to 1.02)<br>(p=0.537)
TC	BMI<21<br>BMI>21	16/94<br>14/110	Ref<br>0.79 (0.39 to 1.63)	-<br>0.533	2/47<br>3/66	0.24 (0.06 to 1.04)<br>**0.26 (0.08 to 0.89)**	0.057<br>**0.031**	0.24 (0.06 to 1.04)<br>0.33 (0.09 to 1.14)	0.22 (-0.49 to 0.94)<br>(p=0.545)
TG	BMI<21<br>BMI>21	16/115<br>19/129	Ref<br>1.12 (0.57 to 2.18)	-<br>0.742	8/45<br>8/75	1.35 (0.58 to 3.16)<br>0.82 (0.35 to 1.92)	0.485<br>0.654	1.35 (0.58 to 3.16)<br>0.74 (0.32 to 1.69)	-0.65 (-2.09 to 0.80)<br>(p=0.382)

High Lipid (+) = 3rd tertile: High Lipid (-) = 1st or 2nd tertile; RERI = relative excess risk due to interaction.

LDL, Low density lipoprotein cholesterol; HDL, High density lipoprotein cholesterol; TC, Total cholesterol; TG, Triglycerides.

Adjusted for age; sex; CCI (Charlson comorbidity index); transplantation; alcoholism; smoking; initial AFB smear; presence of cavitary disease; metformin, statin, and calcium channel blocker use.

Ref = Reference group for the stratified analysis

Supplementary Table 9: Test of Effect modification of lipid levels on mortality by BMI with a cut-off value of 22kg/m2

Type of lipid	BMI cut-off	Mortality						HR (95%CI) # for mortality within each BMI stratum for high lipid (+) compared to (-)	RERI
		High lipid (-)			High lipid (+)				
		n/N	HR (95%CI) #	P-value	n/N	HR (95%CI) #	p-value		
All-cause mortality									
LDL	BMI<22<br>BMI>22	21/87<br>13/49	Ref<br>1.09 (0.55to2.18)	-<br>0.800	4/31<br>4/37	0.49 (0.17 to 1.45)<br>0.42 (0.14 to 1.23)	0.202<br>0.113	0.49 (0.17 to 1.45)<br>0.39 (0.13 to 1.19)	-0.17 (-1.18 to 0.84)<br>(p=0.734)
HDL	BMI<22<br>BMI>22	20/70<br>14/56	Ref<br>0.83 (0.42 to 1.64)	-<br>0.587	4/38<br>2/22	0.31 (0.11 to 0.91)<br>0.28 (0.06 to 1.19)	0.106<br>0.065	0.31 (0.11 to 0.91)<br>0.34 (0.08 to 1.49)	0.14 (-0.62 to 0.89)<br>(p=0.720)
TC	BMI<22<br>BMI>22	32/131<br>17/73	Ref<br>0.98 (0.54 to 1.76)	-<br>0.944	4/57<br>5/56	**0.26 (0.09 to 0.75)**<br>**0.34 (0.13 to 0.87)**	**0.012**<br>**0.024**	0.26 (0.09 to 0.75)<br>0.35 (0.13 to 0.94)	0.09 (-0.59 to 0.78)<br>(p=0.786)
TG	BMI<22<br>BMI>22	31/153<br>23/91	Ref<br>1.19 (0.70 to 2.02)	-<br>0.514	16/59<br>12/61	1.29 (0.71 to 2.33)<br>0.93 (0.48 to 1.80)	0.403<br>0.836	1.29 (0.71 to 2.33)<br>0.78 (0.39 to 1.58)	-0.55 (-1.64 to 0.54)<br>(p=0.324)
Infection related mortality									
LDL	BMI<22<br>BMI>22	14/87<br>7/49	Ref<br>0.88 (0.36 to 2.18)	-<br>0.782	2/31<br>1/37	0.38 (0.09 to 1.67)<br>**0.16 (0.02 to 0.88)**	0.086<br>**0.021**	0.38 (0.09 to 1.67)<br>0.19 (0.02 to 1.51)	-0.09 (-1.16 to 0.96)<br>(p=0.867)
HDL	BMI<22<br>BMI>22	14/70<br>6/56	Ref<br>0.51 (0.19 to 1.33)	-<br>0.167	2/38<br>1/22	0.23 (0.05 to 1.00)<br>0.20 (0.03 to 1.54)	0.050<br>0.122	0.23 (0.05 to 1.00)<br>0.40 (0.05 to 3.35)	0.47 (-0.24 to 1.17)<br>(p=0.197)
TC	BMI<22<br>BMI>22	23/131<br>7/73	Ref<br>0.56 (0.24 to 1.31)	-<br>0.183	2/57<br>3/56	**0.19 (0.04 to 0.79)**<br>**0.29 (0.09 to 0.96)**	**0.023**<br>**0.042**	0.19 (0.04 to 0.79)<br>0.52 (0.13 to 2.02)	0.54 (-0.08 to 1.16)<br>(p=0.082)
TG	BMI<22<br>BMI>22	22/153<br>13/91	Ref<br>1.04 (0.52 to 2.06)	-<br>0.917	10/59<br>6/61	1.24 (0.59 to 2.61)<br>0.73 (0.29 to 1.79)	0.578<br>0.486	1.24 (0.59 to 2.61)<br>0.71 (0.27 to 1.86)	-0.54 (-1.83 to 0.74)<br>(p=0.405)

High Lipid (+) = 3rd tertile: High Lipid (-) = 1st or 2nd tertile; RERI = relative excess risk due to interaction.

LDL, Low Density Lipoprotein Cholesterol; HDL, High Density Lipoprotein Cholesterol; TC, Total Cholesterol; TG, Triglycerides.

#adjusted for Age; sex; CCI- Charlson Comorbidity Index; Transplantation; Alcoholism; smoking; initial AFB smear; presence of cavitary disease; metformin, statin, and calcium channel blocker use

Ref = Reference group for the stratified analysis

Supplementary Table 10: Test of Effect modification of lipid levels on high CRP (>5mg/dL) by BMI with a cut-off value of 20kg/m2

Type of lipid	BMI cut-off	Mortality: High lipid (-) n/N	High lipid (-) Adjusted OR (95%CI) #	High lipid (-) P-value	High lipid (+) n/N	High lipid (+) Adjusted OR (95%CI) #	High lipid (+) p-value	Adjusted OR (95%CI) for high inflammation within each BMI stratum for high lipid (+) compared to (-)	RERI
**High CRP (> 5 mg/dL)**									
LDL	BMI<20	21/34	Ref	-	1/3	0.53 (0.04 to 7.13)	0.631	0.53 (0.04 to 7.13)	-0.32 (-2.05 to 1.42)
	BMI>20	29/45	0.95 (0.35 to 2.58)	0.921	5/20	**0.16 (0.04 to 0.62)**	**0.008**	**0.22 (0.07 to 0.70)**	(p=0.720)
HDL	BMI<20	19/28	Ref	-	3/9	0.23 (0.04 to 1.21)	0.083	0.23 (0.04 to 1.21)	0.32 (-0.44 to 1.09)
	BMI>20	26/43	0.61 (0.20 to 1.77)	0.356	5/17	**0.15 (0.04 to 0.62)**	**0.008**	0.40 (0.13 to 1.25)	(p=0.321)
TC	BMI<20	32/52	Ref	-	2/6	0.35 (0.06 to 2.18)	0.262	0.35 (0.06 to 2.18)	-0.07 (-1.09 to 0.96)
	BMI>20	42/66	0.95 (0.42 to 2.14)	0.900	8/27	**0.24 (0.08 to 0.67)**	**0.007**	**0.26 (0.10 to 0.69)**	(p=0.896)
TG	BMI<20	36/57	Ref	-	9/16	0.86 (0.27 to 2.77)	0.801	0.86 (0.27 to 2.77)	0.24 (-0.86 to 1.34)
	BMI>20	44/78	0.59 (0.28 to 1.24)	0.165	27/44	0.69 (0.29 to 1.65)	0.402	0.97 (0.46 to 2.06)	(p=0.669)
**High WBC (>10 X 10^6/µL)**									
LDL	BMI<20	13/46	Ref	-	0/10	1.0 (1.0 to 1.0)	-	1.0 (1.0 to 1.0)	-0.44 (-1.05 to 0.18)
	BMI>20	15/66	0.75 (0.29 to 1.90)	0.542	6/45	**0.31 (0.09 to 0.99)**	**0.049**	0.38 (0.14 to 1.09)	(p=0.162)
HDL	BMI<20	11/33	Ref	-	2/17	0.26 (0.05 to 1.42)	0.121	0.26 (0.05 to 1.42)	0.031 (-0.47 to 1.09)
	BMI>20	16/63	0.62 (0.23 to 1.69)	0.354	4/37	**0.19 (0.05 to 0.75)**	**0.017**	0.33 (0.11 to 1.03)	(p=0.440)
TC	BMI<20	21/68	Ref	-	2/19	**0.16 (0.03 to 0.85)**	**0.031**	**0.16 (0.03 to 0.85)**	0.37 (-0.18 to 0.93)
	BMI>20	26/99	0.64 (0.30 to 1.35)	0.241	6/61	**0.17 (0.05 to 0.48)**	**0.001**	**0.38 (0.17 to 0.88)**	(p=0.192)
TG	BMI<20	22/80	Ref	-	4/22	0.63 (0.18 to 2.17)	0.463	0.63 (0.18 to 2.17)	0.67 (-0.25 to 1.59)
	BMI>20	24/123	0.61 (0.30 to 1.22)	0.160	19/69	0.91 (0.42 to 1.95)	0.806	1.23 (0.65 to 2.35)	(p=0.153)
**High NL ratio (>4)**									
LDL	BMI<20	14/45	Ref	-	0/10	1.0 (1.0 to 1.0)	-	1.0 (1.0 to 1.0)	-0.20 (-0.68 to 0.27)
	BMI>20	13/63	0.51 (0.19 to 1.35)	0.175	5/41	0.30 (0.09 to 1.07)	0.063	0.71 (0.23 to 2.16)	(p=0.402)
HDL	BMI<20	10/33	Ref	-	4/17	0.75 (0.18 to 3.16)	0.695	0.75 (0.18 to 3.16)	0.33 (-0.83 to 1.48)
	BMI>20	10/61	0.37 (0.12 to 1.14)	0.084	6/34	0.45 (0.13 to 1.57)	0.211	0.81 (0.27 to 2.43)	(p=0.581)
TC	BMI<20	20/65	Ref	-	3/18	0.44 (0.11 to 1.79)	0.249	0.44 (0.11 to 1.79)	0.31 (-0.44 to 1.07)
	BMI>20	21/91	0.49 (0.22 to 1.09)	0.079	7/56	**0.23 (0.08 to 0.65)**	**0.005**	0.46 (0.19 to 1.09)	(p=0.423)
TG	BMI<20	22/78	Ref	-	9/21	2.05 (0.71 to 5.88)	0.183	2.05 (0.71 to 5.88)	-1.13 (-3.36 to 1.09)
	BMI>20	27/114	0.67 (0.33 to 1.35)	0.265	15/66	0.59 (0.26 to 1.33)	0.199	1.10 (0.56 to 2.15)	(p=0.319)

High Lipid (+) = 3rd tertile: High Lipid (-) = 1st or 2nd tertile; RERI = relative excess risk due to interaction. LDL, Low Density Lipoprotein Cholesterol; HDL, High Density Lipoprotein Cholesterol; TC, Total Cholesterol; TG, Triglycerides; B, linear regression co-efficient; SE – Standard error; CRP, C-reactive protein; NL ratio, neutrophil-lymphocyte ratio. #adjusted for Age; sex; BMI- Body mass index; CCI- Charlson Comorbidity Index; Transplantation; Alcoholism; smoking; initial AFB smear; presence of cavitary disease; metformin, statin, and calcium channel blocker use. Ref = Reference group for the stratified analysis

Supplementary Table 11: Test of effect modification of lipid levels on high inflammatory markers by BMI (cut-off value of 21kg/m^2)#

Type of lipid	BMI cut-off	Odds Ratio						OR (95%CI) for high inflammation within each BMI stratum for high lipid (+) compared to (-)	RERI (95%CI) (p-value)
		High lipid (-)			High lipid (+)				
		n/N	OR (95%CI)	P-value	n/N	OR (95%CI)	p-value		
High CRP (> 5 mg/dL)									
LDL	BMI<21<br>BMI>21	24/40<br>26/39	Ref<br>1.15 (0.43 to 3.06)	-<br>0.775	3/7<br>3/16	0.67 (0.12 to 3.69)<br>0.11 (0.03 to 0.51)	0.649<br>0.005	0.67 (0.12 to 3.69)<br>0.19 (0.05 to 0.74)	-0.71 (-2.45 to 1.03)<br>(p=0.422)
HDL	BMI<21<br>BMI>21	23/33<br>22/38	Ref<br>0.52 (0.18 to 1.48)	-<br>0.221	3/13<br>5/13	0.13 (0.03 to 0.60)<br>0.21 (0.05 to 0.87)	0.009<br>0.031	0.13 (0.03 to 0.60)<br>0.66 (0.19 to 2.36)	0.56 (-0.01 to 1.12)<br>(p=0.054)
TC	BMI<21<br>BMI>21	36/60<br>38/58	Ref<br>1.08 (0.49 to 2.40)	-<br>0.844	5/14<br>5/19	0.42 (0.12 to 1.46)<br>0.19 (0.05 to 0.64)	0.175<br>0.007	0.42 (0.12 to 1.46)<br>0.25 (0.08 to 0.78)	-0.32 (-1.39 to 0.76)<br>(p=0.564)
TG	BMI<21<br>BMI>21	42/69<br>38/66	Ref<br>0.64 (0.30 to 1.35)	-<br>0.241	14/25<br>22/35	0.77 (0.29 to 2.03)<br>0.82 (0.34 to 2.01)	0.603<br>0.671	0.77 (0.29 to 2.03)<br>0.97 (0.42 to 2.27)	0.41 (-0.55 to 1.37)<br>(p=0.404)
High WBC (>10 X 10^6/µL)									
LDL	BMI<21<br>BMI>21	14/55<br>14/57	Ref<br>0.99 (0.39to 2.52)	-<br>0.997	3/18<br>3/37	0.58 (0.13 to 2.49)<br>0.20 (0.05 to 0.82)	0.459<br>0.025	0.58 (0.13 to 2.49)<br>0.23 (0.07 to 0.79)	-0.37 (-1.72 to 0.98)<br>(p=0.592)
HDL	BMI<21<br>BMI>21	14/40<br>13/56	Ref<br>0.49 (0.19 to 1.27)	-<br>0.140	3/25<br>3/29	0.24 (0.06 to 0.97)<br>0.17 (0.04 to 0.70)	0.044<br>0.014	0.24 (0.06 to 0.97)<br>0.35 (0.09 to 1.24)	0.44 (-0.16 to 1.05)<br>(p=0.151)
TC	BMI<21<br>BMI>21	23/79<br>24/88	Ref<br>0.76 (0.37 to 1.59)	-<br>0.468	5/33<br>3/47	0.29 (0.09 to 0.94)<br>0.12 (0.04 to 0.46)	0.037<br>0.002	0.29 (0.09 to 0.94)<br>0.32 (0.12 to 0.82)	0.06 (-0.65 to 0.77)<br>(p=0.861)
TG	BMI<21<br>BMI>21	23/100<br>23/103	Ref<br>0.95 (0.47 to 1.91)	-<br>0.879	9/33<br>14/58	1.24 (0.49 to 3.14)<br>1.04 (0.47 to 2.30)	0.656<br>0.932	1.24 (0.49 to 3.14)<br>1.21 (0.46 to 3.19)	-0.15 (-1.51 to 1.22)<br>(p=0.832)
High NL ratio (>4)									
LDL	BMI<21<br>BMI>21	15/52<br>12/56	Ref<br>0.87 (0.43 to 1.75)	-<br>0.700	2/18<br>3/33	2.28 (0.95 to 5.50)<br>0.52 (0.21 to 1.25)	0.065<br>0.700	2.28 (0.95 to 5.50)<br>0.59 (0.17 to 2.06)	-1.64 (-3.81 to 0.53)<br>(p=0.140)
HDL	BMI<21<br>BMI>21	12/39<br>8/55	Ref<br>0.29 (0.09 to 0.87)	-<br>0.527	5/25<br>5/26	0.66 (0.18 to 2.38)<br>0.40 (0.11 to 1.46)	0.527<br>0.164	0.66 (0.18 to 2.38)<br>0.95 (0.29 to 3.18)	0.45 (-0.48 to 1.38)<br>(p=0.349)
TC	BMI<21<br>BMI>21	21/74<br>20/82	Ref<br>0.61 (0.27 to 1.34)	-<br>0.216	6/32<br>4/42	0.62 (0.21 to 1.86)<br>0.18 (0.05 to 0.60)	0.402<br>0.005	0.62 (0.21 to 1.86)<br>0.33 (0.12 to 0.91)	-0.05 (-0.97 to 0.86)<br>(p=0.903)
TG	BMI<21<br>BMI>21	24/96<br>25/96	Ref<br>0.67 (0.34 to 1.35)	-<br>0.265	14/32<br>10/55	2.05 (0.71 to 5.88)<br>0.58 (0.34 to 1.35)	0.184<br>0.199	2.05 (0.71 to 5.88)<br>0.80 (0.38 to 1.69)	-1.13 (-3.36 to 1.09)<br>(p=0.319)

High Lipid (+) = 3rd tertile: High Lipid (-) = 1st or 2nd tertile; RERI = relative excess risk due to interaction.

LDL, Low density lipoprotein cholesterol; HDL, High density lipoprotein cholesterol; TC, Total cholesterol; TG, Triglycerides; B, Linear regression co-efficient; SE, Standard error; CRP, C-reactive protein; NL ratio, neutrophil-lymphocyte ratio.

Adjusted for age; sex; CCI (Charlson comorbidity index); transplantation; alcoholism; smoking; initial AFB smear; presence of cavitary disease; metformin, statin, and calcium channel blocker use.

Ref = Reference group for the stratified analysis
